# Integrative pathway analysis of genome-wide association studies and gene expression data in prostate cancer

**DOI:** 10.1186/1752-0509-6-S3-S13

**Published:** 2012-12-17

**Authors:** Peilin Jia, Yang Liu, Zhongming Zhao

**Affiliations:** 1 Department of Biomedical Informatics, Vanderbilt University School of Medicine, Nashville, TN, USA; 2 Department of Cancer Biology, Vanderbilt University School of Medicine, Nashville, TN, USA; 3 Department of Psychiatry, Vanderbilt University School of Medicine, Nashville, TN, USA; 4 Center for Quantitative Sciences, Vanderbilt University, Nashville, TN, USA

## Abstract

**Background:**

Pathway analysis of large-scale *omics *data assists us with the examination of the cumulative effects of multiple functionally related genes, which are difficult to detect using the traditional single gene/marker analysis. So far, most of the genomic studies have been conducted in a single domain, e.g., by genome-wide association studies (GWAS) or microarray gene expression investigation. A combined analysis of disease susceptibility genes across multiple platforms at the pathway level is an urgent need because it can reveal more reliable and more biologically important information.

**Results:**

We performed an integrative pathway analysis of a GWAS dataset and a microarray gene expression dataset in prostate cancer. We obtained a comprehensive pathway annotation set from knowledge-based public resources, including KEGG pathways and the prostate cancer candidate gene set, and gene sets specifically defined based on cross-platform information. By leveraging on this pathway collection, we first searched for significant pathways in the GWAS dataset using four methods, which represent two broad groups of pathway analysis approaches. The significant pathways identified by each method varied greatly, but the results were more consistent within each method group than between groups. Next, we conducted a gene set enrichment analysis of the microarray gene expression data and found 13 pathways with cross-platform evidence, including "Fc gamma R-mediated phagocytosis" (*P*_GWAS _= 0.003, *P*_expr _< 0.001, and *P*_combined _= 6.18 × 10^-8^), "regulation of actin cytoskeleton" (*P*_GWAS _= 0.003, *P*_expr _= 0.009, and *P*_combined _= 3.34 × 10^-4^), and "Jak-STAT signaling pathway" (*P*_GWAS _= 0.001, *P*_expr _= 0.084, and *P*_combined _= 8.79 × 10^-4^).

**Conclusions:**

Our results provide evidence at both the genetic variation and expression levels that several key pathways might have been involved in the pathological development of prostate cancer. Our framework that employs gene expression data to facilitate pathway analysis of GWAS data is not only feasible but also much needed in studying complex disease.

## Background

Prostate cancer is the most common cancer diagnosed in men in the USA [1]. During the past decades, tremendous efforts have been made to understand the underlying molecular mechanisms of prostate cancer in both genetic components and at the transcriptional level. As of 3/15/2012, a total of 18 genome-wide association (GWA) studies (17 for prostate cancer and 1 for prostate cancer mortality) have been reported and deposited in the NHGRI GWAS Catalog database [2]. These studies revealed more than 70 single nucleotide polymorphisms (SNPs) linked to prostate cancer. Additionally, gene expression studies augmented by microarray technologies have been conducted to identify disease candidate genes; such efforts were made before the adoption of popular GWA studies and continue to accumulate comprehensive gene expression profiles for prostate cancer. The well-designed genomics projects in each domain have helped investigators to generate massive amount of genetic data, presenting new opportunities to interrogate the information revealed in each single domain and to explore combined analyses across platforms. Recently, mapping genetic architecture using both genome-wide association studies and microarray gene expression data has become a promising approach, especially for the detection of expression quantitative trait loci (eQTLs) [3-5]. Alternatively, a systems biology approach that integrates genetic evidence from multiple domains has its advantages in the detection of combined genetic signals at the pathway or network level. Such an approach is urgently needed because results among different genomic studies of complex diseases are often inconsistent and numerous genomic datasets for each complex disease have already made available to investigators.

We designed this project to analyze GWAS and microarray gene expression data in prostate cancer at the gene set level, aiming to reveal gene sets that are aberrant in both the genetic association and gene expression studies. Gene set (e.g., biological pathway) analysis of large scale *omics *data has recently been proposed as a complementary approach to single marker or single gene based analyses [6-8]. It builds on the assumption that a complex disease might be caused by changes in the activities of functional pathways or functional modules, in which many genes could be coordinated, yet each individual gene might play only a weak or modest role on its own. According to this assumption, investigation of a group of functionally related genes, such as those in the same biological pathway, has the potential to improve power. Pathway analysis may also provide further insights into the mechanisms of disease because they highlight underlying biological relevance.

Over the past several years, a series of methods have been published for gene set analysis. These methods can be broadly categorized into two groups based on their testing hypotheses [7-9]: 1) the competitive null hypothesis (Q1), which tests whether the genes in a gene set show similar association patterns with the disease compared to genes in the rest of the genome; and 2) the self-contained null hypothesis (Q2), which tests whether the genes in a gene set are associated with the disease. Currently, specific methods were developed to investigate either the GWAS data [10-12] or microarray gene expression [13,14] individually, while other methods were created that are applicable to both platforms with slight adaptations [13,15,16]. For example, the Gene Set Enrichment Analysis (GSEA) method from the Q1 group was initially developed for gene expression data [13] and has recently been adapted to GWAS [16], followed by its various extensions (e.g., GSEA-SNP [17] and *i*-GSEA4GWAS [18]). Unlike gene expression data for which both the technologies and methods have matured, GWAS data analysis presents numerous challenges, including testing millions of SNPs per sample and subsequent multiple test corrections, complex local linkage disequilibrium (LD) structures, and heavy computational duties due to thousands of samples, especially in permutation analysis. Several methods were specifically designed for GWAS data by taking these features into account, such as the Association List Go AnnoTatOR (ALIGATOR) [10] in the Q1 group, and the Adaptive rank truncated product statistic (ARTP) [12], the SNP Ratio Test (SRT) [19], and the *t*-statistic in mixed model [20] in the Q2 group. Aside from the essential differences in hypothesis testing, each of these methods has its own strengths and weaknesses in dealing with complex genetic and phenotype data for disease association, requiring careful design in practice.

In this study, we conducted a comprehensive pathway analysis of a prostate cancer GWAS dataset utilizing four representative methods from the two hypothesis testing schemes. We further analyzed the pathways (or gene sets) enriched in a public microarray gene expression dataset using the GSEA method. Both platforms (GWAS and gene expression) were leveraged on the pathway collection annotated by the KEGG database as well as several specially designed gene sets. Our comparison within the GWAS platform showed that the significant pathways detected by each method varied substantially, but the consistency within the same hypothesis method group was greater than those between method groups. Furthermore, we combined the pathway results in GWAS and microarray gene expression data using the Fisher's method. A total of 13 KEGG pathways were found as significant in the combined analysis, confirming our hypothesis that changing activities in pathways indeed show cross-platform consistency. The results in this combined analysis might be more reliable; thus, they warrant further experimental investigation.

## Materials and methods

### Datasets

The GWAS prostate cancer data used in this study is part of the Cancer Genetic Markers Susceptibility (CGEMS) study [21]. We downloaded the data from the National Center for Biotechnology Information (NCBI) dbGaP [22] through approved access. Approximately 550,000 SNPs were genotyped using two types of chips: Illumina HumanHap300 (Phase 1A) and Illumina HumanHap240 (Phase 1B). The data included 1172 prostate cancer patients and 1157 controls of European ancestry from the Prostate, Lung, Colon and Ovarian (PLCO) Cancer Screening Trial [23]. We filtered SNPs based on the following quality check criteria: locus call rates (< 90%), minor allele frequency (MAF < 0.05), and monomorphic status across array types [24]. Samples that were genotyped by both HumanHap300 and HumanHap240 were selected, and those with missing genotype data > 0.1 were excluded. The cleaned data included a total of 506,216 SNPs and 2243 samples (Table 1). We used the basic allelic test for association test of SNPs with prostate cancer. The genomic inflation factor was 1.03. Throughout this study, wherever applicable, we mapped a SNP to a gene if it was located within the gene or 20 kb from the boundary of the gene [6].

**Table 1:** Summary of genotyping (GWAS) data and microarray gene expression data.

	GWAS	Microarray gene expression
Source	CGEMS [24]	GEO: GDS2547 [25,26]
# of features	506,216 SNPs	10,595 genes
# of samples	Cases:1146 Controls: 1097	Cases: 64 Controls: 75
Phenotype	Prostate cancer	Subset 1 (17, normal prostate tissue) Subset 2 (58, normal prostate adjacent to tumor) Subset 3 (64, primary prostate tumor)
Platform	Illumina HumanHap300 and HumanHap240	Affymetrix Human Genome U95C array

CGEMS: The Cancer Genetic Markers of Susceptibility (CGEMS) project. GEO: Gene Expression Omnibus.

For gene expression data, we selected a public microarray dataset from the NCBI Gene Expression Omnibus (GEO) database with a similar phenotype and appropriate sample size (GDS2547) [25,26]. A total of 64 primary prostate tumor samples and 75 controls (17 normal prostate tissue samples and 58 normal prostate samples adjacent to tumor) were included as our working dataset [26]. A standard processing procedure was implemented, including quantile normalization of the samples, *t*-test for differential expression, and multiple testing correction. For genes with multiple probe sets, we computed the median value to represent the gene. A summary of the above two datasets is available in Table 1.

### Gene set selection

The Molecular Signatures Database (MSigDB) [13] is a database that collects gene sets from various sources, including online pathway databases, publications in PubMed, and the knowledge of domain experts. Among these collections, we chose to use the pathways from the KEGG database [27] in the C2 category. To avoid too many or too few genes to be considered in each pathway analysis, we only included the pathways whose sizes were between 5 and 250 genes in our following analysis. This process resulted in a total of 181 qualified pathways.

In addition to the publicly available pathways, we defined several knowledge-based gene sets for our analysis. First, we manually collected a list of candidate genes for prostate cancer downloaded from the Human Prostate Gene Database (PGDB) [28], a well-curated and integrated database for prostate and prostatic diseases. We retrieved 129 genes and denoted them as one gene set, namely the PGDB gene set.

**Figure 1:**
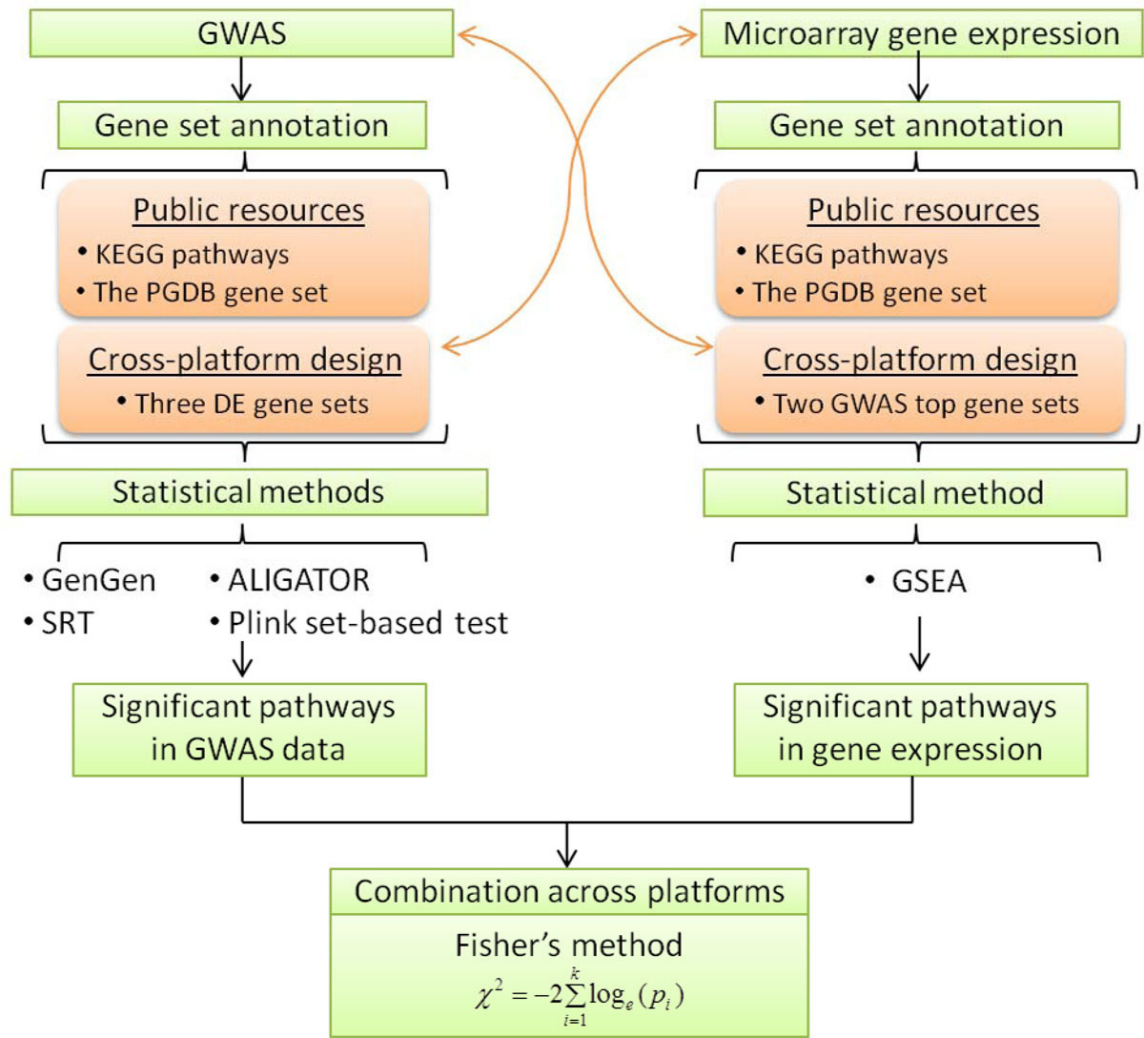
**Workflow of the integrative pathway analysis**. PGDB: Human Prostate Gene Database; DE: differentially expressed; ALIGATOR: Association List Go AnnoTatOR; SRT: SNP Ratio Test; GSEA: Gene Set Enrichment Analysis.

Second, for pathway analysis of the GWAS data, we defined 3 additional gene sets from the microarray gene expression data in order to perform cross-platform evaluation. Genes that were differentially expressed with *FDR *< 0.05 in *t*-test and with log2 ratio (LR) under three different thresholds (i.e., 1, 1.5, and 2) between case and control samples were extracted to form three expression-based external gene sets (Table 2). They were named DEG_LR_1 (LR > 1 or LR < -1), DEG_LR_1.5 (LR > 1.5 or LR < -1.5), and DEG_LR_2 (LR > 2 or LR < -2); here, DEG denotes differentially expressed genes. These gene sets were defined based on gene expression information and were included only in the pathway analysis of the GWAS data (Figure 1). In summary, for the pathway analysis of the GWAS data, we had 185 gene sets: 181 KEGG pathways, the PGDB gene set, and 3 gene sets derived from gene expression.

**Table 2:** Description of additional gene sets.

Gene set	Size	Description
PGDB	129	Genes extracted from the PGDB database [28]
GWAS_Top30	30	Top 30 genes with the smallest association *P*-values in GWAS.
GWAS_TopP-4	69	Top 69 genes with association *P *< 10^-4 ^in GWAS
DEG_LR_1	165	DEGs in gene expression data with *FDR *< 0.05, and absolute log2 ratio (LR) > 1
DEG_LR_1.5	130	DEGs in gene expression data with *FDR *< 0.05, and absolute LR > 1.5
DEG_LR_2	13	DEGs in gene expression data with *FDR *< 0.05, and absolute LR > 2

DEG: differentially expressed gene (DEG). FDR value was based on *t*-test. More details of gene sets are provided in the text.

Third, for pathway analysis of gene expression data, aside from the KEGG pathways and the PGDB gene set, we similarly defined additional gene sets from GWAS data analysis results. The first one included the top 30 genes ranked by their gene-wise *P*-values in association with prostate cancer, while the second one included the genes whose gene-wise *P*-values were < 10^-4 ^(69 genes). We defined these two sets as GWAS_Top30 and GWAS_TopP-4. As a result, for the pathway analysis of microarray gene expression data, we had a total of 184 gene sets: 181 KEGG pathways, the PGDB gene set, the GWAS_Top30, and the GWAS_TopP-4 (Table 2).

### Pathway analysis methods for GWAS data

Previous studies have proposed many approaches for gene set analysis of GWAS data [6-8]. However, so far, no single method has been shown to outperform the other methods in the analysis of different GWAS data sets. To avoid the potentially biased application of any one algorithm, we chose four representative methods to perform a comprehensive analysis in this study. Two of these methods belong to the Q1 group of competitive hypothesis, namely, the GSEA method for GWAS data implemented in the software GenGen [16,29] and the method ALIGATOR [10]. The other two methods, the SRT and the Plink set-based test, are from the Q2 group of self-contained hypothesis testing.

The GSEA algorithm was initially developed for gene expression data analysis [13] and has been recently extended to GWAS data [16,29]. The software GenGen [30] is one of the toolkits that implement the GSEA algorithm. In brief, the following steps are taken when GenGen is applied. First, it defines gene-wise statistical values. Given multiple SNPs mapped to a gene region, a popularly adopted approach is to use the maximum statistical value of all SNPs within or near the gene region to represent its association significance. For example, the SNP with the maximum χ^2 ^value is chosen as the representative SNP, and the corresponding χ^2 ^value is assigned as the gene-wise statistical value for the gene. Next, all genes are ranked according to their χ^2 ^values. Third, for each pathway, an enrichment score (ES) is calculated as the maximum departure of the genes in the pathway from zero. Finally, the significance of the ES for each pathway is estimated through the permutation of sample labels. In GWAS, this is done by swapping the case and control status to keep the LD structure among SNPs/genes. The analysis is then executed in each set of permutation data. A normalized ES (NES) and an empirical *P*-value are typically calculated for each pathway.

ALIGATOR [10] tests the overrepresentation of gene sets within genes that contain significantly associated SNPs from GWAS data. It takes the association *P*-values of single SNPs as analysis units and preselects criterion to define significant SNPs (e.g., *P *< 0.05). Genes that contain significant SNPs are counted, but each gene is only counted once regardless of how many significant SNPs are involved in it. Instead of permuting phenotypes, ALIGATOR permutes SNPs. In each permutation, SNPs are randomly selected from the pool, and once a new SNP is selected, the number of genes that contain significant SNPs in the selected collection is counted and compared with the corresponding number in the real case. The random selection process continues until the number of significant genes targeted by the selected SNPs is the same as in the original study. Finally, an empirical *P*-value is computed for each pathway based on the permutation data.

The SNP Ratio Test (SRT) [19] builds on the ratio of significant SNPs in a pathway and estimates the significance of the ratio utilizing permutation data. Similar to the process used by ALIGATOR, a cutoff value is preselected to distinguish significant SNPs from non-significant ones. In this study, we used 0.05. The significance of each pathway is estimated by an empirical *P*-value through permutation on phenotypes.

The Plink set-based test [31] provides an average statistical test of sets of SNPs. Given a query pathway with the SNPs mapped to the genes in this pathway, the set-based test determines groups of SNPs based on their local LD structure and selects the "current" best SNP in every step. Briefly, it first selects the best SNP and removes the other SNPs within the same LD, defined by r^2 ^values (e.g., r^2 ^> 0.5). In the remained SNPs, the set-based test again searches for the best SNP and removes highly related SNPs. Then, the process is repeated until *P*-values of the remaining SNPs are below a pre-defined cutoff. The average of the statistical values of the selected SNPs is obtained for each pathway and permutation of phenotype labels is performed to compute an empirical *P*-value for each gene set.

### Pathway analysis methods for microarray gene expression

The GSEA algorithm in gene expression data analysis was first introduced by Subramanian et al. [13,15] and has become a popular tool for interpreting gene expression data at the pathway level. The underlying algorithm for GSEA is essentially the same as described above for GWAS data, except that the gene-wise statistical value is a signal to noise ratio that is computed based on gene expression data. A detailed description can be found in the original publication [13]. In our application, we used the software GSEA downloaded from reference [32]. Multiple testing correction using the false positive rate (*FDR*) is incorporated to adjust gene set *P*-values.

### Fisher's method

Fisher's method combines multiple probabilities from independent tests of the same hypothesis and generates one combined statistic (χ^2^) using the following formula:

$$\chi^{2}=-2\overset{k}{\underset{i=1}{\sum }}{log}_{e}\left(p_{i}\right)$$

where *p_i _*is the *P*-value for the *i*^th ^hypothesis test, and *k *is the number of tests being combined [33]. Theoretically, χ^2 ^has a chi-square distribution with *2 k *degree of freedom when all *p_i _*values are independent.

In this study, we used the Fisher's method to combine individual nominal *P*-values obtained from GWAS and microarray gene expression analyses for eligible pathways in both platforms. A combined *P*-value was then computed for each pathway to show the consistency of its association with prostate cancer from different types of genomic data.

## Results

Figure 1 illustrates the workflow. We applied four methods for the prostate cancer CGEMS GWAS data and one method for the prostate cancer microarray gene expression data. Table 3 lists the parameters used for each method. It also summarizes the significant pathways identified in each analysis scenario. Among the 4 methods used for GWAS data, GenGen is threshold-free, while the three other methods require a pre-defined cutoff value to distinguish significant SNPs. In these cases, we used cutoff value 0.05 (Table 3). We performed permutation 1000 times in each of the four cases (GenGen, SRT, Plink set-based test, and GSEA) by swapping case/control labels. For ALIGATOR, because the resampling unit is SNP (~550,000 SNPs in our dataset), we permuted a larger number of times, i.e., 10,000 times (Table 3). Because the signals from GWAS data could be weak and the coherence across platforms are presumably also weak, we set up two tiers of criteria to define significant pathways. The tier one criterion is relatively loose and was based on nominal *P*-values, i.e., pathways with nominal *P *< 0.01 were selected. The tier two criterion was built on *FDR*, i.e., pathways with *FDR *< 0.2 were selected (Table 3). Note that instead of the traditional cutoff *P*-value 0.05, we used *FDR *< 0.2 such that marginally significant pathways would not be overlooked and an appropriate number of pathways could be derived.

**Table 3:** Parameters and summary of the significant pathways by each pathway analysis method.

Method	Parameter(s)	# pathways (*P *< 0.01)	# pathways (*FDR *< 0.2)	The PGDB gene set	Other external gene sets
GenGen	*π *= 1000	4	3	N	N
ALIGATOR	*π *= 10,000, *P *= 0.05	0	0	N	N
SRT	*π *= 1000, *P *= 0.05	3	0	N	N
Plink set-based test	*π *= 1000, *r^2 ^*= 0.5, *P *= 0.05, *max *= 5	15	15	Y	N
GSEA	*π *= 1000	5	7	N	N

This table lists the parameters used in GenGen, ALIGATOR, SRT, the Plink set-based test, and the Gene Set Enrichment Analysis (GSEA). The third and forth columns contain the number of pathways selected by tier one criterion (nominal *P *< 0.01) and tier two criterion (*FDR *< 0.2), respectively. The fifth and sixth columns indicate whether the method could identify the PGDB gene set or other additional gene sets (more details available in main text).

*π*: number of permutations.

### Pathway analysis of CGEMS prostate cancer GWAS data

For GWAS data, the Plink set-based test generated the largest number of significant pathways among the four methods, regardless of tier one or tier two criterion. It identified 15 significant pathways, including the PGDB gene set; however, these significant pathways did not include the three gene sets defined by expression data. GenGen identified 4 pathways that were nominally associated with prostate cancer, three of which were significant at *FDR *< 0.2. However, none of the external gene sets, including the PGDB gene set, were found by GenGen to be significant. SRT found 3 nominally significant pathways using tier one criterion, but none passed the multiple testing correction using tier two criterion (Table 3). ALIGATOR essentially found no significant pathway.

Among the 15 significant pathways identified by the Plink set-based test (Table 4), seven belong to the "Human Diseases → Cancers" group in the KEGG maps. These pathways are: "chronic myeloid leukemia (hsa05220)," "small cell lung cancer (hsa05222)," "endometrial cancer (hsa05213)," "thyroid cancer (hsa05216)", "bladder cancer (hsa05219)," "acute myeloid leukemia (hsa05221)," and "colorectal cancer (hsa05210)." Notably, the Plink set-based test is the only method that could identify the PGDB gene set as significant. The PGDB gene set was ranked as the 14^th ^most significant gene set, with a nominal *P*-value = 0.004 and *FDR *= 0.053. Because the PGDB gene set contains prostate cancer candidate genes collected from various type of evidence, especially functional gene studies [28], and GWA studies are designed as essentially hypothesis-free (not specifically for a disease or a set of disease genes), the successful identification of this gene set to be significantly enriched within an independent GWAS dataset is promising, suggesting an appropriate analysis might be able to unveil genetic components in GWA studies.

**Table 4:** Significant pathways (*FDR *< 0.01) detected by the Plink set-based test.

Pathway (KEGG ID)	*P*	*FDR*
Jak-STAT signaling pathway (hsa04630)*	0.001	0.043
Chronic myeloid leukemia (hsa05220)	0.001	0.043
Small cell lung cancer (hsa05222)	0.001	0.043
TGF-beta signaling pathway (hsa04350)	0.002	0.043
Endometrial cancer (hsa05213)	0.002	0.043
Thyroid cancer (hsa05216)*	0.002	0.043
Bladder cancer (hsa05219)	0.002	0.043
Acute myeloid leukemia (hsa05221)	0.002	0.043
Cell cycle (hsa04110)	0.003	0.043
Wnt signaling pathway (hsa04310)	0.003	0.043
Fc gamma R-mediated phagocytosis (hsa04666) **^†^**	0.003	0.043
Regulation of actin cytoskeleton (hsa04810) **^†^**	0.003	0.043
Colorectal cancer (hsa05210)	0.003	0.043
**The PGDB gene set**	0.004	0.053
ErbB signaling pathway (hsa04012)	0.008	0.099

*Overlap with SRT.

^†^Overlap with GSEA.

The other significant pathways identified by the Plink set-based test also showed strong relevance. Interestingly, the most significant pathway, "Jak-STAT signaling pathway (hsa04630)," is the underlying signaling mechanism for a wide range of cytokines and growth factors. The roles of JAK/STAT in prostate cancer have been well studied in many reports [34-36]. Among the 155 genes involved in this pathway, 67 had nominally significant gene-wise *P*-values in the association test (*P *< 0.05), 6 of which had gene-wise *P*-value < 1 × 10^-3 ^(Table 5). This observation suggests the importance of this pathway involved in the pathology of prostate cancer.

**Table 5:** List of "Jak-STAT signaling pathway (hsa04630)" genes with gene-wise association *P *< 0.001 in CGEMS prostate cancer GWAS data.

Gene symbol	Most significant SNP	**Chr**.	Position (bp)	Genomic region	*P*
*MYC*	rs7837688	8	128539360	Intron	4.96 × 10^-7^
*CSF2RB*	rs909486	22	37323988	Intron	1.85 × 10^-4^
*PIAS1*	rs11071981	15	68416575	Intron	2.63 × 10^-4^
*IL2RA*	rs3118470	10	6101713	Intron	3.29 × 10^-4^
*SPRY2*	rs1999494	13	81000505	Intron	4.01 × 10^-4^
*LEP*	rs12538332	7	127839654	Intron	5.24 × 10^-4^

Chr.: chromosome. bp: base pair.

### Pathway analysis of gene expression data

For gene expression data, a total of 184 gene sets were eligible for analysis using the GSEA method with 1000 permutations. Five pathways had nominal *P*-values less than 0.05, while seven pathways were identified by applying an *FDR *cutoff 0.2. All seven pathways were from the KEGG annotations. No external gene sets (GWAS-derived gene sets) were found to be significant (Table 6).

**Table 6:** Significant pathways in prostate cancer microarray gene expression data detected by the GSEA method.

Pathway (KEGG ID)	*ES*	*NES*	*P*	*FDR*
Fc gamma R-mediated phagocytosis (hsa04666)*	0.645	1.809	< 0.001	0.131
Focal adhesion (hsa04510)	0.482	1.658	0.004	0.176
Dilated cardiomyopathy (hsa05414) **^†^**	0.672	1.905	0.004	0.101
Hypertrophic cardiomyopathy (HCM) (hsa05410) **^†^**	0.619	1.740	0.009	0.122
Regulation of actin cytoskeleton (hsa04810)*	0.464	1.714	0.009	0.128
Leukocyte transendothelial migration (hsa04670)	0.594	1.760	0.011	0.163
Arrhythmogenic right ventricular cardiomyopathy (ARVC) (hsa05412) **^†^**	0.663	1.760	0.012	0.123

ES: enrichment score. NES: normalized enrichment score.

*Overlap with the Plink set-based results.

**^†^**Overlap with the GenGen results.

### Comparison among methods and platforms

**Figure 2:**
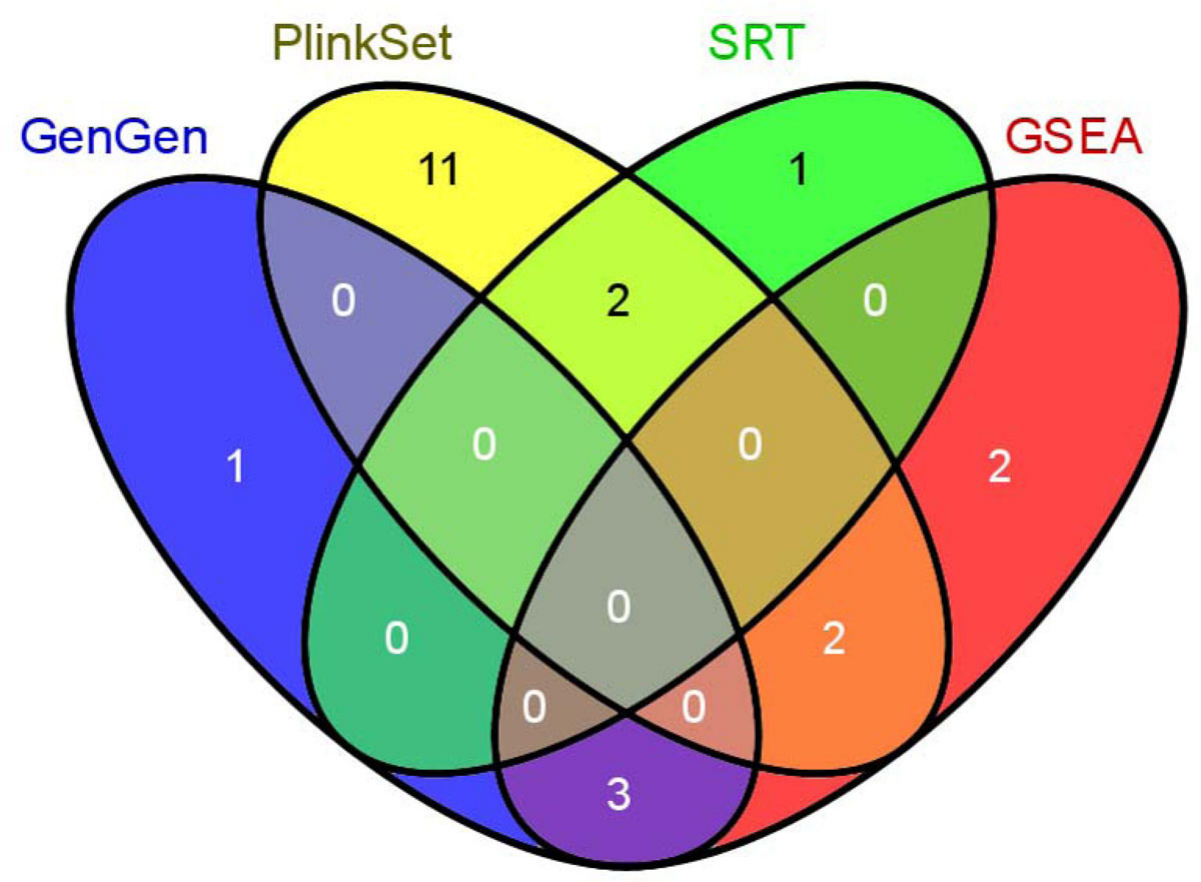
**Comparison of the significant pathways found in genome-wide association studies and microarray gene expression datasets by different methods**. This Venn diagram shows the comparison of significant pathways detected using the prostate cancer GWAS dataset using three methods: GenGen, Plink set-based test (PlinkSet), and the SNP Ratio Test (SRT), and the significant pathways found in microarray gene expression data by the Gene Set Enrichment Analysis (GSEA) method. Note that ALIGATOR identified no significant pathways and was not included in the comparison.

To explore the overlap among the significant pathways identified by each method, we compared four result sets and drew a Venn diagram (Figure 2). These pathways included: 1) 4 pathways by GenGen (GWAS); 2) 15 pathways by the Plink set-based test (GWAS); 3) 3 pathways by the SRT (GWAS); and, 4) 7 pathways by GSEA (gene expression). Note that for each method, we selected the pathways passing either tier one or tier two criterion so that all detected pathways were included. ALIGATOR generated no significant pathway and, thus, was not included in this comparison.

No pathways were identified by at least 3 methods. Seven pathways were identified by at least two methods. Among them, three pathways, i.e., "arrhythmogenic right ventricular cardiomyopathy (ARVC) (hsa05412)," "hypertrophic cardiomyopathy (HCM) (hsa05410)," and "dilated cardiomyopathy (hsa05414)," were detected by both GenGen (GWAS data) and GSEA (expression data). Two pathways, "Jak-STAT signaling pathway (hsa04630)" and "thyroid cancer (hsa05216)," were detected by the Plink set-based test and SRT, both in the GWAS data. Another two pathways, "Fc gamma R-mediated phagocytosis (hsa04666)" and "regulation of actin cytoskeleton (hsa04810)," were identified by both the Plink set-based test in the GWAS data and GSEA in the gene expression analysis.

### Combined analysis of pathways

For the 148 common pathways that were eligible for both the Plink set-based analysis of GWAS data and GSEA of microarray gene expression data, we combined their nominal *P*-values derived from each dataset based on the Fisher's method. Thirteen pathways were found to have combined *P*-values < 0.01 (Table 7).

**Table 7:** Significant pathways (*P *< 0.01) by a combined analysis using the Plink set-based test and GSEA.

Pathway (KEGG ID)	GSEA	Plink	*P*_combined_	*FDR*
Fc gamma R-mediated phagocytosis (hsa04666)	< 0.001	0.003	6.18 × 10^-8^	9.15 × 10^-6^
Regulation of actin cytoskeleton (hsa04810)	0.009	0.003	3.34 × 10^-4^	2.47 × 10^-2^
Jak-STAT signaling pathway (hsa04630)	0.084	0.001	8.79 × 10^-4^	3.56 × 10^-2^
Dilated cardiomyopathy (hsa05414)	0.003	0.024	9.63 × 10^-4^	3.56 × 10^-2^
Small cell lung cancer (hsa05222)	0.266	0.001	2.45 × 10^-3^	7.27 × 10^-2^
Hypertrophic cardiomyopathy (HCM) (hsa05410)	0.009	0.051	4.20 × 10^-3^	9.62 × 10^-2^
Cell cycle (hsa04110)	0.251	0.003	6.16 × 10^-3^	9.62 × 10^-2^
Arrhythmogenic right ventricular cardiomyopathy (ARVC) (hsa05412)	0.012	0.068	6.46 × 10^-3^	9.62 × 10^-2^
Chronic myeloid leukemia (hsa05220)	0.843	0.001	6.80 × 10^-3^	9.62 × 10^-2^
Bladder cancer (hsa05219)	0.422	0.002	6.80 × 10^-3^	9.62 × 10^-2^
Wnt signaling pathway (hsa04310)	0.297	0.003	7.14 × 10^-3^	9.62 × 10^-2^
TGF-beta signaling pathway (hsa04350)	0.508	0.002	8.01 × 10^-3^	9.66 × 10^-2^
Axon guidance (hsa04360)	0.016	0.074	9.18 × 10^-3^	9.66 × 10^-2^

In general, the combined results of the Fisher's method highly ranked the pathways that were found to be consistently significant across multiple studies. For example, three of the top four pathways were nominally significant in both GWAS and expression data: the pathways of "Fc gamma R-mediated phagocytosis (hsa04666)" (*P*_GWAS _= 0.003, *P*_expr _< 0.001, and *P*_combined _= 6.18 × 10^-8^), "regulation of actin cytoskeleton (hsa04810)" (*P*_GWAS _= 0.003, *P*_expr _= 0.009, and *P*_combined _= 3.34 × 10^-4^) and "dilated cardiomyopathy (hsa05414)" (*P*_GWAS _= 0.003, *P*_expr _= 0.024, and *P*_combined _= 9.63 × 10^-4^). The pathway "Jak-STAT signaling pathway (hsa05216)," which was the most significant in GWAS data analysis but was not significant in gene expression data (*P*_GWAS _= 0.001, *P*_expr _= 0.084, and *P*_combined _= 8.79 × 10^-4^), was ranked third by the Fisher's method. These results further indicate that there are indeed pathways that are disturbed at different levels, e.g., genetically (germline mutations) or by transcriptional dosages. Therefore, these pathways are more likely to be involved in the mechanisms of prostate cancer. Based on this integrative pathway analysis, we defined these 13 pathways as candidate pathways for prostate cancer.

We further checked the genes in the candidate pathways for their overlap with two well-curated candidate gene sets for cancer: the gene list specifically collected for prostate cancer and the general one for all cancer types from the Cancer Gene Census (CGC) [37]. Note that the PGDB gene set was not included in the candidate pathways. As shown in Additional file 1, 30 genes from the prostate cancer candidate pathways were also collected by the prostate cancer database (PGDB), while 80 were collected by CGC as known cancer genes. The results here indicate the signals are enriched in these candidate pathways.

## Discussion

In this study, we utilized four pathway analysis methods to test the association of the KEGG pathways with prostate cancer in the CGEMS GWAS dataset. The four methods, namely GenGen, ALIGATOR, SRT and Plink set-based test, represent two groups of hypothesis testing methods for the pathway analysis of GWAS data, i.e., the competitive and self-contained groups. In addition, we incorporated a microarray gene expression dataset with similar phenotypes for prostate cancer and performed pathway analysis using GSEA. Genetic evidence from the GWAS and expression data naturally formed an independent validation of each other and at two different domain levels (association signals and differential gene expression). Straightforward examination of the overlapping pathways between the two dataset platforms, as well as a combined analysis using the Fisher's method, highlighted several pathways that are significantly associated with prostate cancer. These results supported the rationale of our motivation to combine cross-platform information at the gene set level, and they shed new light on the candidate pathways that are likely involved in prostate cancer.

In the pathway analysis of GWAS data, results varied greatly among different methods. To generate an objective comparison, we defined a relatively loose criterion based on nominal *P*-values, i.e., the tier one criterion (nominal *P*-value < 0.01), and a more strict criterion based on adjusted *P*-values after multiple testing correction, i.e., the tier two criterion (*FDR *< 0.2). In terms of the number of significant pathways, the Plink set-based test generated the most (15 significant pathways by both tiers of criteria), followed by GenGen (4 by tier one and 3 by tier two), SRT (3 by tier one and none by tier two), and ALIGATOR (none by either tier of criterion). For the shared pathways, overlap is quite limited among the different methods, with only two pathways shared by the Plink set-based test and SRT (Figure 2). The results from GenGen did not share any pathways with the other three methods. This comparison reflects the current challenges of the pathway analysis of GWAS. Furthermore, the limited overlap among the different methods is not surprising, as each method has its own evaluation focus of disease associations. As we mentioned above, both GenGen and ALIGATOR belong to the "competitive" method group, while the Plink set-based test and SRT belong to the "self-contained" group [7,8]. Indeed, results by the Plink set-based test and SRT shared two nominally significant pathways, although no overlap with those by either GenGen or ALIGATOR in the "competitive" group. Nevertheless, different methods may have their own advantages and disadvantages in determining different types of pathways and specific phenotype data of the GWA studies [38].

In this study, we uniquely recruited several special gene sets in the pathway analysis. Among those six external gene sets, except the PGDB gene set, none were found to be significant in the cross-platform evaluation. That is, none of the three gene sets defined by differentially expressed genes were identified to harbour significant association information in GWAS data, and none of the two gene sets consisting of top associated genes in GWAS data were found to be significant in the gene expression data. This observation suggests that a straightforward selection of candidate gene sets primarily based on one domain might be difficult to replicate in another domain, even though in the same disease phenotype. Rather, functional gene sets such as pathways are more likely to be found as significant at different levels of the biological systems, such as from the level of genetic components to transcriptional changes. This point further supports our design of a comparative analysis of pathways, which represent dynamic biological processes that, if disturbed, may cause the disease.

Among the candidate pathways for prostate cancer, the most promising one is "Jak-STAT signaling pathway (hsa04630)," which mediates signaling that starts with the cytokines, signals through Jak-STAT mediated activities, and finally regulates downstream gene expression [39]. Mutations in JAKs and constitutive activation of STAT have been observed in a variety of diseases, including cancers [40]. Interestingly, we observed two receptor genes that have low *P*-values in the CGEMS GWAS data: *CSF2RB *(gene-wise *P *= 1.85 × 10^-4^) and *IL2RA *(gene-wise *P *= 3.29 × 10^-4^). In the Jak-STAT signaling pathway, cytokine receptors mediate signaling from extracellular to intracellular upon the binding of cytokines to their extracellular domains. This process occurs at the most upstream of the overall signaling transduction; therefore, cytokine receptors play important roles in this pathway. Both CSF2RB and IL2RA belong to the class I receptor family and are associated with Jak docking [41]. In both of these genes, their most significant SNPs are located in the intronic region rather than within their amino acid coding regions. Since the association signals indicate there are possible causal mutations in the genomic region, future investigation of the true causal functional SNPs that tag with these significant SNPs, and their roles in prostate cancer, is warranted. Moreover, we found several other genes with small association *P*-values in this pathway: gene *PIAS1 *(*P *= 2.63 × 10^-4^), an inhibitor of STAT, and its two downstream genes, *MYC *(*P *= 4.96 × 10^-7^) and *SPRY2 *(*P *= 4.01 × 10^-4^).

## Conclusions

In summary, we conducted an integrative pathway analysis of GWAS data and microarray gene expression data augmented by knowledge-based gene set annotations. We explored four representative methods for the pathway analysis of GWAS data, among which the Plink set-based test generated the most sensible set of significant pathways both statistically and in biological interpretation. Together with the results from gene expression data for the same disease, we combined the results from different platforms and identified 13 candidate pathways for prostate cancer. This analysis framework confirmed the concept of a combined pathway analysis utilizing information from different genomics platforms, and it can be extended to the analysis of genomics data in other complex disease.

## Competing interests

The authors declare that they have no competing interests.

## Authors' contributions

PJ prepared the data, carried out the data analysis, and contributed to the writing of the manuscript. YL prepared the data, carried out the data analysis, and contributed to the writing of the manuscript. ZZ conceived and designed the study, provided the data, participated in the project discussion, and contributed to the writing of the manuscript. All authors read and approved the final manuscript.

## Supplementary Material

Additional file 1 **Details of significant pathways by the Plink set-based test, GSEA or the combined analysis (*FDR *< 0.2)**. Click here for file
